# Renal Delivery of Pharmacologic Agents During Machine Perfusion to Prevent Ischaemia-Reperfusion Injury: From Murine Model to Clinical Trials

**DOI:** 10.3389/fimmu.2021.673562

**Published:** 2021-07-06

**Authors:** Rossana Franzin, Alessandra Stasi, Marco Fiorentino, Simona Simone, Rainer Oberbauer, Giuseppe Castellano, Loreto Gesualdo

**Affiliations:** ^1^ Department of Emergency and Organ Transplantation, Nephrology, Dialysis and Transplantation Unit, University of Bari Aldo Moro, Bari, Italy; ^2^ Department of Nephrology and Dialysis, University Clinic for Internal Medicine III, Medical University Vienna, Vienna, Austria; ^3^ Nephrology, Dialysis and Transplantation Unit, Advanced Research Center on Kidney Aging (A.R.K.A.), Department of Medical and Surgical Sciences, University of Foggia, Foggia, Italy

**Keywords:** renal ischemia/reperfusion, machine perfusion, hypothermic perfusion, normothermic perfusion, DCD − donation after cardiac death, ECD − expanded donor criteria, complement system, senolytic agents

## Abstract

Donor organ shortage still remains a serious obstacle for the access of wait-list patients to kidney transplantation, the best treatment for End-Stage Kidney Disease (ESKD). To expand the number of transplants, the use of lower quality organs from older ECD or DCD donors has become an established routine but at the price of increased incidence of Primary Non-Function, Delay Graft Function and lower-long term graft survival. In the last years, several improvements have been made in the field of renal transplantation from surgical procedure to preservation strategies. To improve renal outcomes, research has focused on development of innovative and dynamic preservation techniques, in order to assess graft function and promote regeneration by pharmacological intervention before transplantation. This review provides an overview of the current knowledge of these new preservation strategies by machine perfusions and pharmacological interventions at different timing possibilities: in the organ donor, *ex-vivo* during perfusion machine reconditioning or after implementation in the recipient. We will report therapies as anti-oxidant and anti-inflammatory agents, senolytics agents, complement inhibitors, HDL, siRNA and H2S supplementation. Renal delivery of pharmacologic agents during preservation state provides a window of opportunity to treat the organ in an isolated manner and a crucial route of administration. Even if few studies have been reported of transplantation after *ex-vivo* drugs administration, targeting the biological pathway associated to kidney failure (i.e. oxidative stress, complement system, fibrosis) might be a promising therapeutic strategy to improve the quality of various donor organs and expand organ availability.

## Introduction

**Figure 1:**
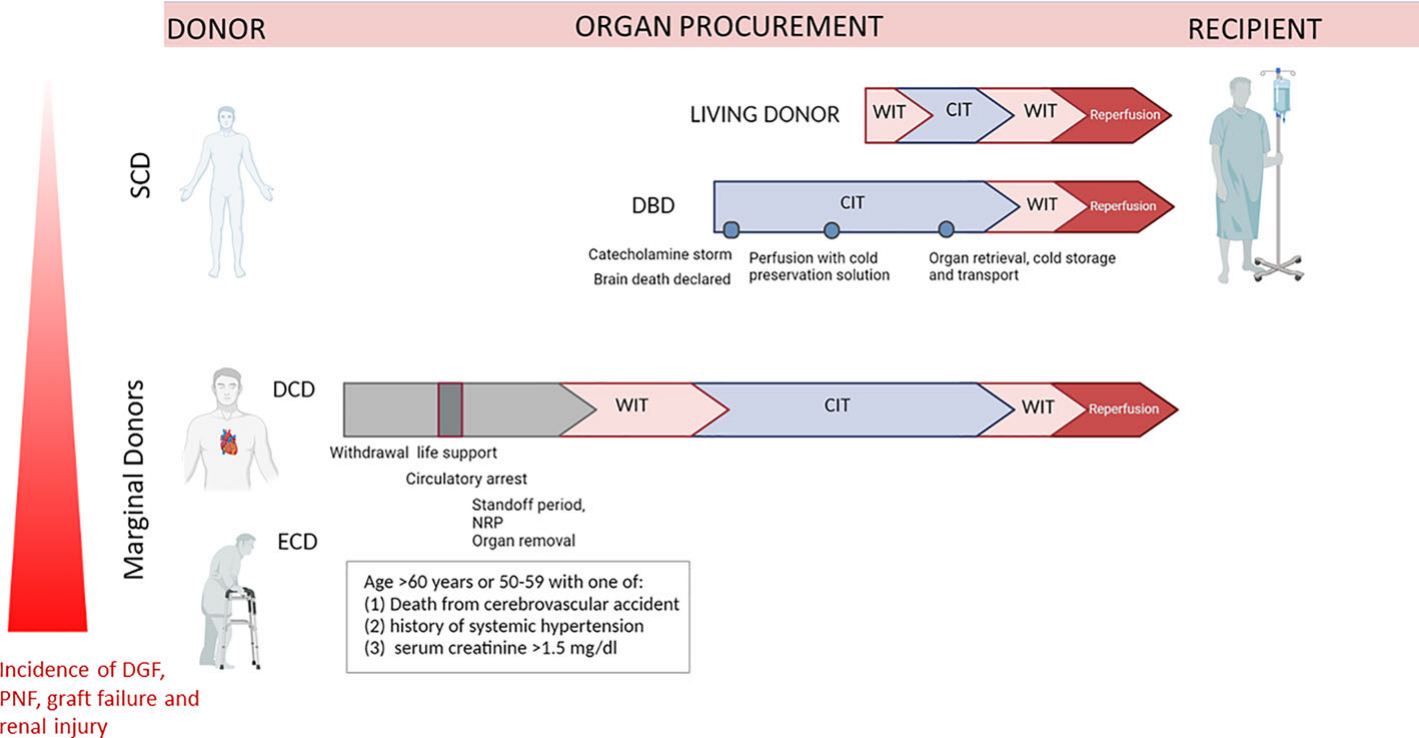
Representation of different ischemia types exposure of organs retrieved from Living Donor (LD), DBD, DCD and ECD. Organ retrieved by DCD and ECD, or DCD+ECD donors are subjected to longer warm ischemia time due to cardiocirculatory arrest, whereas in DBD surgical preparation and perfusion with cold preservation solution is initiated immediately after cerebral death. DCD, donation after circulatory death; DBD, donation after brain death; ECD, Expanded criteria donors; WIT, warm ischemia time; CIT, cold ischemia time; SCD, Standard Criteria Donors.

Kidney transplantation represents the best treatment for patient with End-Stage Kidney Disease (ESKD) (1). Renal transplantation is widely recognized as the most optimal long-term treatment option in terms of mortality, life quality, and cost when compared to haemodialysis. However, the growing disparity between the number of organ donors and the demand for kidney transplantation has become a great drawback for the nephrology community. At the time of writing, in Europe more than 11000 individuals are registered in kidney waiting list (https://statistics.eurotransplant.org), with a median wait-time for first transplant of 3.6 years. In renal transplant candidates, annual mortality ranges from 5 to 10% worldwide, with sharp increase in the older population (>50 years) (2). Specifically, almost 50% of patients older than 60 years of age who are renal transplant candidates in the United States die before receiving a renal graft (3). The severe organ shortage has prompted the acceptance of strategies to increase the pool of donors. One emerging approach is the expansion to include more marginal organs, as occurred for the utilization of kidneys after circulatory death (DCD) and expanded criteria donors (ECD) (4, 5). However, the use of ECD and DCD kidneys still requires a huge price to pay related to increased incidence of primary non-function (PNF), delayed graft function (DGF) and reduced long-term graft survival (**Figure 1**). To improve renal outcomes, research has focused on development of preservation techniques, in order to treat kidneys, assess their quality and function, promote recovery and repair by therapeutic intervention before transplantation. In the last decade, exciting advancements have been made in the development of dynamic technologies of preservation that imply the circulation of a perfusate either at hypothermic (4-10°C), subnormothermic (20-25°C) or normothermic (37°C) temperatures (6–9). Multiple devices and systems have been developed, become commercially available, CE marked and are routinely used in different centres. Machine perfusion can represent a bridge between the donor and recipient and may provide a platform for direct, non-systemic drug treatment of the kidney.

This review focuses on the recent understanding of molecular mechanisms and factors responsible of renal Ischemia/reperfusion injury, strategies of interventions and advances in the application of innovative therapies to improve kidney quality during perfusion.

## From Marginal Kidneys to ECD and DCD Classification

In living donation (LD), organs are recovered from healthy individuals and rapidly transplanted within a coordinated surgical environment, resulting in a short ischaemia time and long-term graft survival. Brain Death donors (DBD) are often associated with several complications (i.e. related to catecholamine and cytokine storm), moreover this transplantation is characterized by longer cold ischaemia times than for LD procedure (10) (**Figure 1**). However, both living donors and DBD (Donation after Brain Death) can be included in the wide classification of Standard criteria donors (SCD). By contrast, organs from DCD and ECD donors are exposed to huge physiological and haemodynamic changes, including substantially longer warm ischaemia, due to cessation of cardiac activity, but also to hormone dysregulation, pro-inflammatory responses, oxidative stress and increased complement activation. Additionally, chronic pre-existing comorbidities are often associated with immune activation while the donor is still alive.

The term ECD was introduced for the first time in 1997 by Kauffman et al. and completely substituted the broad definition of marginal kidneys (11, 12). Nowadays, ECD organ are defined by deceased donors aged 60 years or older or aged 50–59 years with at least two of the three following criteria: cerebrovascular cause of death, terminal serum creatinine higher than 1.5mg/dl or history of hypertension (11, 13). In 2019, in Europe, 30% of potential donors are ECD and a similar percentage of these kidneys are discarded annually. It’s well recognized that kidneys from ECD correlated with approximately 2-fold increased risk of DGF, acute rejection, and graft loss (14, 15) (**Figure 1**).

Regarding the classification of donation after circulatory death (DCD) donors, also referred to as nonheart-beating donors (NHBD) or death after cardiac death donors, it was regulated by the First International Workshop in Maastricht. First of all, DCD donation takes place after declaration of death based on cardiorespiratory criteria in contrast to DBD that required neurological criteria (16–18). In addition, while in DBD organs are perfused until the moment of preservation, with almost absent first warm ischemia time, organs from DCD donors underwent warm ischemia between circulatory arrest and the start of organ perfusion (**Figure 1**). Four categories of DCD donors have been classified, distinguished in two main type: uncontrolled (uDCD), referred to patients with unexpected cardiac arrest with unsuccessful resuscitation and controlled DCD (cDCD), in which the moment of the arrest of life-sustaining therapy can be planned, and therefore with known length of warm ischemia time. Today, DCD donors are mainly referred to category III-controlled DCD donors. In the last fifteen years, country that registered the highest DCD activity were United Kingdom, Spain, Russia, the Netherlands, Belgium and France (19). As expected, graft from cDCD donors showed better post‐transplant outcomes that patients who received kidneys from uDCD donors. Despite initial controversial ethical and legal discussion, DCD is becoming progressively established and performed in Europe, contributing to increase the pull of organs available and giving adequate graft outcomes. What should be clear, is that simple cold storage, the actual gold standard of organ preservation, is not sufficient to optimally preserve organs from DCD donors. New organ preservation methods to better protect and recondition DCD organs are thus being developed including normothermic regional perfusion (NRP) to resuscitate abdominal organs *in situ* prior to cold flushing or *ex-vivo* machine perfusion preservation after organ procurement (20, 21).

## From Brain Death to Renal Ischemia Reperfusion: Molecular Mechanisms of Graft Damage Brain Death, Cold Ischemia Time and Donor Characteristics

**Figure 2:**
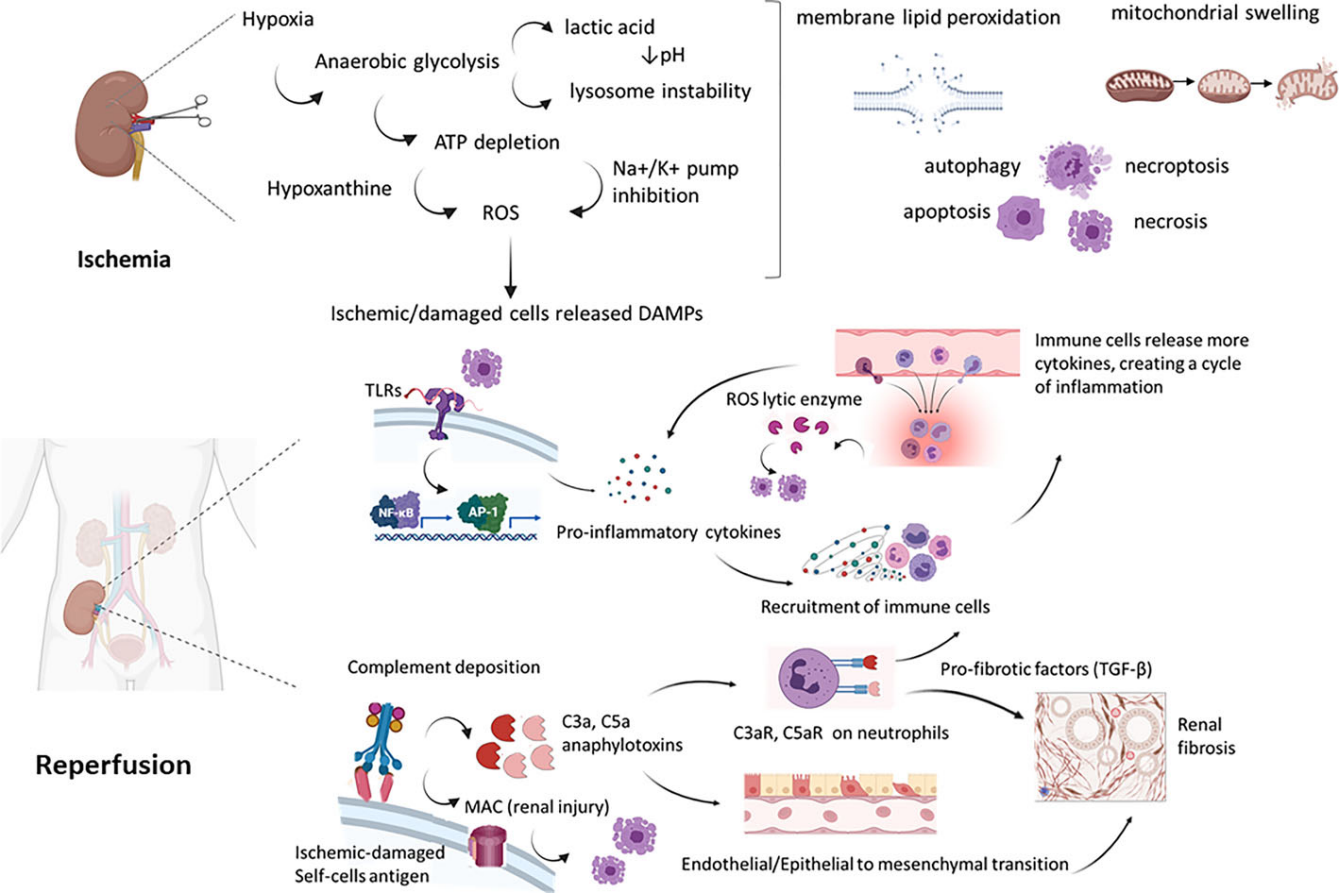
Molecular mechanisms of renal IRI. During ischemia, the lack of oxygen and substrates led to inhibition of oxidative phosphorylation, thereby to ATP depletion. From a side this led to an anaerobic lactic acid-associated glycolysis, with pH decrease and lysosome lytic enzyme release. From the other, the blockade of pump Na/K activated proteases and phospholipases, leading to increased Ca++ level. Furthermore, ATP produced in aerobic tissues is lysed into AMP, adenosine, inosine and hypoxanthine. Hypoxanthine is metabolized by xanthine oxidase in ischemic tissues, in a reaction that uses molecular oxygen (O_2_) and release toxic ROS as intermediate products. During reperfusion, DAMP released by ischemic damaged kidney cells are recognized by PRR as TLR on immune cells but also on endothelial cells leading to increased gene expression of pro-inflammatory cytokines that recruited and activated leucocytes. These cells released more cytokines, in an amplification loop culminating into ROS release by macrophages and neutrophils, interstitial infiltrates and kidney damage. Ischemic damaged cells can activate complement system (by Collectin-11, MBL) that result in anaphylotoxins C3a and C5a generation and MAC-mediated cell injury. These acute processes have been linked to early renal fibrosis development by the process of EndMT, EMT and PMT (Endothelial to mesenchymal transition, Epithelial to mesenchymal transition and Pericytes to mesenchymal transition). TLR, Toll Like receptor; MAC, membrane associated complex; DAMP, Damage-associated molecular patterns; PRR, Pattern Recognition Receptors; ROS, reactive oxygen species.

Several mechanisms and risk factors have been proposed to contribute to IRI in kidney transplantation (22) (**Figure 2**). Although IRI may also occur in kidney transplantation from living donors, this condition is more severe in organs from deceased donors. Compared to graft biopsy of living donors, specimens of DBD were characterized by an increased transcriptional activation of complement components, acute-phase proteins and chemokines (23). Hruba et al. confirmed these findings in recipients with graft biopsy performed early after transplantation comparing organs from SCD and ECD. Authors showed upregulation of transcripts related to inflammation, wounding and defence responses, complement and coagulation and cytokine–cytokine receptor interaction pathways (24). Brain death is usually associated with a hyperactivation of the sympathetic system in order to maintain an adequate cerebral perfusion: moreover, cytokines and growth factors released by brain injury may induce renal ischemia (25). In addition, hypotensive episodes occurring during hospitalization requiring the use of vasoconstrictor agents may further contribute to kidney hypoperfusion. Prolonged cold ischemia time (CIT) severely affects oxygen and nutrients supply to tissues and may induce and aggravate IRI; after graft removal, a kidney is stored in cold solution to preserve the viability of its cells, but this process cannot completely prevent cellular injury (26). There is a progressive detrimental effect of CIT on transplant outcome, with 90% survival at 1 year for organs transplanted within 20 hours and a lower percentage for organs transplanted at > 30 hours (relative risk 1.9). In a UK study, protracted CIT was associated with poor kidney graft survival in recipients of DCD kidneys. On the other hand, higher donor age correlated with earlier graft failure for both DCD and DBD donor kidneys (27). Whereas the impact of CIT on early post-transplant period is well defined, the link with long-term graft survival is controversial. Notably, in recipients of ECD organs included in the Scientific Registry of Transplant Recipients, overall graft loss was not significantly different between recipients with higher CIT and paired donor recipients with lower CIT (28). Finally, in a study including 3829 adult recipients of a first heart-beating deceased-donor kidney transplantation, Debout et al. showed a proportional relationship between each additional hour of CIT and the risk of graft failure, as it was higher when CIT was longer than 36h (8% *vs* 4% when CIT was less than 16h at 1 year, 36% *vs* 20% at 10 years) (29), suggesting the importance of minimizing CIT to improve short- and long-term outcomes of kidney transplantation.

### Complement System in Renal Transplantation: Strategies of Inhibition in the Donor and During Preservation

The activation of immune system plays a central role in all transplantation phases (10, 14, 30, 31).

The stepwise process of transplantation consists of consecutive events that can affect the graft, including brain or cardiac death in deceased donors, unavoidable ischemia/reperfusion injury, preservation procedure, post-transplantation rejection and other non-immunological insults such as drug toxicity, diabetes or hypertension (10). During all these stages, various immune responses can potentially induce graft injury and contribute to premature loss of kidney function. Particularly, early innate immune response appeared more detrimental than major histocompatibility complex, as emerged by improved outcomes from HLA- unmatched living donor transplantation compared to HLA- matched deceased donor transplantation (10, 32).

Complement has a critical role in immunological and inflammatory processes before, during and after transplantation. Indeed, complement can exacerbate graft damage, leading to early fibrosis and a premature aging, thus resulting in a gradual deterioration of function (33–36).

Briefly, complement can be activated by three pathway: the classical pathway (CP), lectin pathway (LP), and the alternative pathway (AP) (37–39). The CP is initiated by C1q binding to antigen-antibody complexes, thereby inducing a cascade *via* C2 and C4 cleavage, leading to the production of CP C3 convertase (C4b2b). In the LP, mannose-binding lectin (MBL) ficolins or collectin-11, bind to their MBL-associated serine proteases (MASP) and similarly to CP, activate C4b2b convertase. The AP is activated by hydrolysis of C3 to C3(H2O). Factor B is recruited and cleaved by factor D to form the AP C3 convertase (C3bBb). The factor properdin, the only positive regulator of complement system, can stabilize the C3bBb convertase. The C3 convertases formed by the different pathways are responsible for a low-grade cleavage of C3, thereby forming C3b. C3b mediates opsonization or binds to the C3 convertase to form the C5 convertase. The C5-convertases cleave C5 into C5a and C5b, then this became the substrate for following attachment of C6, C7, C8, and C9, leading to membrane attack complex (MAC) generation. The MAC complex induces the formation pores in the membrane of pathogens and damaged self-cells, thus promoting cell lysis. Finally, complement promotes a systemic inflammation by release of C3a and C5a anaphylotoxins, able to induce neutrophils chemotaxis (**Figure 2**).

Complement activation can occur already in waitlisted patients for kidney transplantation particularly during hemodialysis and attributed the contact of systemic complement to biomaterial surfaces inside the circuit tubing and filter membranes (40, 41). Moreover, complement activation in transplant candidates could be the consequence of complement related disease as IgA nephropathy (IgAN), C3 glomerulopathy, membranoproliferative glomerulonephritis type I, atypical haemolytic uraemic syndrome (aHUS) (10, 34, 42, 43).

As demonstrated in human preclinical studies the type of organ affects the role of complement activation. Deceased donors, particularly DCD and DBD donors, have higher level of complement activation as assessed by increased level of C3d, C4d, C5a, Bb or soluble C5b-9 (14, 44–46). Moreover, grafts from DCD donors were more prone to develop acute rejection in the recipient (46, 47).

Brain death exacerbates the detrimental effects of complement activation, and experimental complement inhibition by CR2-Crry in a mouse cardiac transplant model significantly reduced myocardial injury and prolonged graft survival (48).

Furthermore, as demonstrated by Burk et al., multiple trauma occurring in the donor results in a severe “complementopathy”, characterized by immediate activation, consumption, and dysfunction of the complement cascade, that may have consequences in transplantation perspective (49). In addition, also the C3 allotype of the donor (i.e C3S and C3F) has been associated to renal graft survival. Damman et al. demonstrated the protective role of C3F allotype in a cohort of DCD donors that was associated to better outcome in the recipient (50).

After organ procurement, the detrimental role of complement in renal IRI has been extensively investigated by experimental studies using C3–, C5–, and C6–deficient murine model (51–54), pre-clinical porcine studies (55, 56) and clinical trials (57–59).

An emerging therapeutic strategy to arrest upstream complement activation is C1-esterase-inhibitor (C1- INH), an endogenous serine protease inhibitor of classical- and lectin pathway (60, 61). Pre-clinical studies with C1-INH in the deceased donor showed promising results.

In a rat model of brain death, high-dose C1-INH treatment significantly improved renal function and reduced local and systemic inflammation, as assessed by lower serum creatinine level, decreased pro-inflammatory gene expression and serum IL-6 respectively (14, 62).

To evaluate the effect of C1-INH (CINRYZE) as a donor pre-treatment strategy to decrease systemic inflammation and decrease the incidence of DGF in ECD, is currently the aim of phase I, randomized trial (NCT02435732) that is expected to be completed in May 2022. A similar study is ongoing in non-human primate model (Fernandez L. The Role of Complement Inhibition in Expanded Criteria Kidney Transplantation http://grantome.com/grant/NIH/R01- AI110617-03 #5R01AI110617-03) (63). The effect of intraoperative administration of C1-INH (Berinert) to prevent DGF was evaluated by Jordan et al. in 105 recipients of DCD kidneys (61). Even though, C1-INH treatment did not reach the primary end-point on DGF incidence, were found important reductions in need for dialysis and improvements in long-term allograft. Recently, the trial has been updated in the long-term outcome definition, showing that Berinert treatment is associated with a lower incidence of graft failure (64).

In a pig model of renal IRI injury, the treatment with C1-INH conferred protection not only blocking C4d, factor B, C5b-9 deposition but also reducing infiltrating cells, significantly modulating early fibrosis by limiting number of myofibroblasts through endothelial-to-mesenchymal (EndMT), pericytes-to-myofibroblasts (PMT) transitions (56, 65, 66) leading to a reduction of chronic graft fibrosis also after 3 months from transplantation (55). Strikingly, C1-INH modulated the occurrence of premature renal aging of the graft (35, 67, 68).

In the context of renal transplantation, no data are available regarding the supplementation of C1-INH in perfusion solutions. In a porcine extracorporeal liver reperfusion, Bergamaschini et al. that supplemented C1-INH in the perfusate after 8 h of cold ischaemia. The major findings were a reduced C3 activation both at plasma and local level, a drop of total serum haemolytic activity, with lower degree of inflammatory cell infiltration and tissue damage (69).

These results suggest that adding C1-INH to the preservation solution may be useful tool to selectively shut down early complement activation, thereby limiting tissue injury during the reperfusion, without completely “extinguish” the recipient immune system.

Next to the use of complement inhibitors in the donor, other studies evaluated the effect of complement blocking during dynamic perfusion treatment.

sCR1 can inhibit classical and alternative pathways by binding C3b and C4b and resulting in C3 and C5 convertase blockade (58, 70). In a recent model of renal IRI and in a study of an *ex-vivo* normothermic perfusion, Hameed AM et al. tested the administration of combination of sCR1 plus αCD47Ab, a blocking monoclonal antibody able to ameliorates thrombospondin (TSP)-1 mediated IRI signaling, including inhibition of nitric oxide and promotion of oxidative stress (71). Their main conclusion was that αCD47Ab was shown to be most protective as established also by NMP treatment of a porcine DCD model.

Another compound derived from sCR1, the APT070, also known as Mirococept was tested in preservation solutions (14, 72). In an interesting study, after perfusion with Mirococept, rat donor kidneys were exposed to 16 h of cold storage (73), then kidneys were transplanted into syngeneic recipients. Compared to control-cold stored renal allografts, APT070 perfused renal grafts had more than 2 fold higher survival rates.

The clinical translation of these rodents’ analysis recently led to a multicentre randomized controlled trial, in which Mirococept is administered *ex vivo* to deceased donor kidneys. The trial, called EMPIRIKAL, aims to evaluate the efficacy of Mirococept in reducing the incidence of DGF in renal transplants from deceased donors (74, 75) and provided evidences on safety and feasibility of this new drug.

Although these preliminary data warrant further validation, these observations suggest that complement-targeted regulation before, during and after organ retrieval could modulate aberrant inflammation, regulating the IRI cytokine storm thus limiting renal damage and improving the outcome of transplantation.

## Oxidative Stress and Mitochondrial Dysfunction in I/R Injury

The extent of renal dysfunction and loss of parenchymal integrity within transplanted organ is influenced by both I/R injury and mitochondrial dysfunction that are the main triggers of oxidative stress (76). During transplant surgical procedure, both clamping and section of renal artery for organ explant in the donor and later arterial anastomosis in the recipient might determine the start of a form of AKI recognized as DGF (77, 78).

In ischemic condition, the drop in blood flow is sudden and the anaerobic metabolism prevails due to a severe decrease of oxygen in all compartments of renal parenchyma (78–80). This anaerobic metabolism is not able to meet the increasing energy demands of aerobic tissue and low oxygen concentrations impair mitochondrial oxidative phosphorylation, reducing ATP production (81). In order to compensate the decrease of ATP level and maintain cellular energy-dependent processes, the lactate-dependent ATP production is activated but this mechanism generates intracellular acidosis that alters intracellular pH and contributes to cellular dysfunction and damage (81, 82) (**Figure 2**).

ATP depletion reduces the activity of the Na+/K+ATPases, promoting an increase of Na and water influx that induce edema and alteration of intracellular electrolytes homeostasis (83). This disequilibrium stops the activity of Na/Ca^+2^ antiporter membrane protein to pump Ca^2+^ out of the cell, causing an intracellular Ca^+2^ overload (76, 83, 84). Intracellular Ca^2+^ levels are further augmented by the inhibition of Ca^2+^ reuptake into the endoplasmic reticulum due to the ATP depletion. Since the activation of lactate-dependent ATP pathway causes lowering of pH, the Na+/H+ antiporter membrane protein pumps protons out of the cells in an attempt to correct the intracellular pH (82, 84).

All these processes contribute to increase intracellular Ca^2+^ levels that consequently enhance the activation of calcium dependent proteases such as calpains that exert their detrimental function during the reperfusion phase when pH was normalized (85).

The increased intracellular levels of Na and Ca^2+^ trigger mitochondria to internalize Ca^+2^ in their matrix. This accumulation modifies cytochrome c activity and enhance Reactive Oxygen Species (ROS) generation (86). In addition, Ca^2+^ accumulation sensitize and predispose mitochondrial transition pore (mPTP) opening in the reperfusion phase (86) (**Figure 2**).

During hypoxic condition, the activity of antioxidant enzymes, such as superoxide dismutase (SOD), catalase (CAT) and glutathione peroxidase (GSH-Px) is strongly impaired causing an exacerbated production of ROS when blood circulation is restored (87).

Therefore, the reestablishing of blood supply in reperfusion phase enhances several mechanisms that are more detrimental for renal parenchyma and include the burst of ROS, calcium overload, opening of the MPT pore, endothelial dysfunction and activation of coagulation and inflammatory response (88, 89).

The ROS burst and mPTP opening cause an influx of water and solute that enhances mitochondrial swelling and the disruption of the outer membrane with the release of cytochrome C in the cytosol (90). The presence of cytochrome C in the cytosol induces the activation of pro-apoptotic caspase-3 which drives cells to apoptosis (90).

In addition, ROS induce direct damage to cellular macromolecules such as proteins, lipids and DNA, which consequently enhance cells to activate death mechanisms (90). Several evidences suggest that NADPH oxidase plays a central role in mediating oxidative stress and DNA damage and might contribute to progressive renal damage in this setting (91).

Considering the differences of oxygenation among several regions of renal parenchyma, it is obviously that the response of each cell depends on their location within the kidney (92). As well known, the renal outer cortex has high oxygen reserve and renal tubular epithelial cells could resist very well to hypoxic damage if the ischemic period is not too long. Differently tubular cells located in inner medulla are more susceptible to oxygen decrease, since they have an elevate consumption of ATP due to their involvement in reabsorption function. Papillary cells are located in hypoxic region of renal parenchyma and they have an anaerobic metabolism and could resist to reduced oxygen delivery (92).

The principal consequences of I/R injury are associated to profound alterations of renal epithelial cells that include brush border loss, cytoskeletal changes and rapid loss of surface markers and peculiar transport properties that enhance death mechanisms (93). All these functional and structural changes induce epithelial cell desquamation and the appearance of cellular debris aggregates in the tubular lumen (93). Recently, emerging evidences underline that oxidative stress may promote epigenetic modifications of DNA inducing the acquirement of a senescent phenotype that could be the priming of chronic renal damage (68, 94).

Endothelial cells are also involved in this context and are the first target of I/R injury (95, 96). These cells loss their peculiar functions and acquired dysfunctional phenotype increasing renal damage. They are not only the target of this injury, but they are also the principal key players in the pathogenesis of renal damage (97). Recently, our group demonstrated in a swine model of I/R injury, that these cells survived and became myofibroblasts participating in early renal fibrosis (65). Along vascular compartment, pericytes seemed to be involved in the pathophysiology of renal IRI increasing inflammatory cells infiltration and participating in fibrotic process (66). Therefore, all the cells respond to I/R injury as described above and influenced graft outcomes.

Several studies underline the correlation between I/R injury, oxidative stress and graft outcomes (98, 99). Indeed, La Manna G. et al. observed in a cohort of thirty patients that transplant recipients with a significant decrease in IL-6 plasma levels and DNA oxidation and fragmentation had a better recovery of renal function at 6 months after transplantation (100). Moreover, the measurement of cytokine levels confirmed that low levels of oxidative stress correlate to better outcome in kidney allografts (100).

New several polymorphisms in genes involved in oxidative stress have been associated with DGF, allowing better understanding of DGF pathophysiology and suggesting a possible explanation of the different outcomes between individuals in post-transplant course (101, 102). Azmandian J. et al. demonstrated that donors and recipients’ Glutathione S-transferases (GSTs) polymorphism was associated with increased serum creatinine, high levels of lipid peroxidation and increased risk of DGF (101). In accordance, a recent study highlighted the association between recipients’ subunit of NAD(P)H-oxidase polymorphism and DGF occurrence (102).

Given the strong link between oxidative stress, especially in cadaveric donors and poor transplantation outcomes, several studies have investigated the use of antioxidant therapy in kidney transplantation (103). Long C et al. examined the effects of Oleanolic acid (OA) preconditioning in a rat model of I/R injury (104). OA is a natural compound, with known antioxidant, anti−inflammatory, and anti−apoptotic properties. In this experimental study, the authors demonstrated that animal pre-treated with OA for 15 days presented better recovery of renal function and reduced pro-inflammatory cytokines (104) (**Table 1**).

**Table 1:** Pharmacological treatment in transplantation field to prevent IR injury.

Indication	Drug	Study design	Results	References
		Preclinical studies		
***Renal I/R injury***	OA	Animal model: Rats were administered with OA (12.5, 25 and 50 mg/kg) for 15 consecutive days prior to bilateral renal I/R induction.	decreased levels of BUN, sCr, KIM-1 and LDH; decreased levels of MDA, increased activities of superoxide dismutase, catalase and glutathione peroxidase, and increased GSH levels; decreased levels of proinflammatory cytokines and increased of anti-inflammatory cytokine.	(104)
	Leutoline	Animal model: male Swiss albino mice were pre-treated with luteolin (100 mg/kg body weight) seven consecutive days before I/R induction.	significant reduction in the level of TNF-α, IL-1β, and IL-6; preservation of renal tissue and reduction of apoptotic cells	(105)
	NACand ATOR	Animal model: pre-ischemic administration of NAC and/or ATOR (24h before I/R) followed by I/R injury in rats	lower MPO and higher GPx activity in NAC, ATOR, and NAC+ATOR group versus I/R group; lower rate of tubular ischemic lesions in NAC+ATOR versus I/R group	(106)
	C5a siRNA	Animal model: Mice were injected with 50 μg of C5aR siRNA 2 days before induction of ischemia.	reduced BUN and sCr; recovery of renal function; reduced inflammation	(107)
	C3 and Caspase-3 siRNA	Animal model: *in vivo* gene silencing by hydrodynamic injection with C3 and caspase 3 siRNAin mice, before I/R injury.	reduced BUN and sCR; recovery of renal function; reduced mortality	(108)
	CD40 siRNA	Animal model: injection of siRNA anti-CD40 in rodent warm and cold ischemia models	modulation of local and systemic inflammation	(109)
	HDL	Animal model:HDL (80 mg/kg, intravenous) was administered to male Wistar rats 30 min before bilateral renal ischemia for 45 min followed by reperfusion for up to 48 h.	improved renal function; reduced inflammation; reduced endothelial dysfunction; reduced lipid peroxidation and oxidative stress injury	(110)
	Quercitin	Animal model:42 Sprague-Dawley rats were divided into three groups: control, I/R and I/R+quercetin (I/R+Q) . I/R + Q rats were treated with quercetin (50 mg/kg intraperitoneal) 1 h prior to the induction of ischemia.	decreased tissue malondialdehyde (MDA) and increased glutathione (GSH) levels; reduction of apoptotic and p53-positive cells, NF-κB and eNOS expression levels	(111)
***Renal I/R injury / EX vivo perfusion /Transplantation***	Metformin	Animal model: metformin preconditioning and postconditioning during *ex vivo* normothermic machine perfusion (NMP) of rat and porcine kidneys affect I/R injury	both metformin preconditioning and postconditioning can be done safely and improved rat and porcine kidney quality.	(112)
	Heparin	Animal model: brain-dead porcine donors and murine kidneys during CS	amieloration of endothelial function; improved renal function	(113)
	Heparin	Animal model: Brain death pigs. Kidneys (matched pairs; n = 6 + 6) were preserved for 20 hours by HMP during which 50 mg heparin conjugate was added to one of the HMP systems (treated group)	recovery of renal function; reduced inflammation and preservation of renal parenchyma	(114)
	Quercitin	*In vitro* study: proximal tubular epithelial cells were preincubated for three hours with bioflavonoids and cold stored in University of Wisconsin (UW)- or Euro-Collins (EC)-solution for 20 hours	increased cell viability; reduced lipid peroxidation.	(115)
	Quercitin+ Sucrose	Animal model: porcine model of renal autologous transplantation. Left kidney grafts were divided in 3 groups: Cold Storage (CS) preservation for 24 hours; CS preservation for 22 hours and hypothermic oxygenated perfusion (HOPE) with CS/MP-UW solution for 2 hours; CS preservation for 22 hours and hypothermic oxygenated perfusion (HOPE) with CS/MP-UW solution with Quercitin and Sucrose added to the solution.	amieloration of renal function; reduced oxidative stress and parenchymal damage.	(116)
	Naked Caspase-3 siRNA	Animal model: Intravenous injection of 0.9 mg siRNA and right-uninephrectomy; left kidney was autotransplanted for 2 weeks.	reduced inflammation; amelioration of renal function.	(117)
	H2S	Animal model: bilateral nephrectomy rats underwent renal transplantation with kidneys from donor rats that were flushed with cold solution or cold solution plus 150 μM NaHS (H2S group).	reduced renal injury; reduced oxidative stress; reduced Inflammation.	(118)
	H2S	Animal model: bilateral nephrectomy rats underwent renal transplantation with kidneys from donor rats that were flushed with cold solution or cold solution plus 150 μM NaHS (H2S group).	reduced renal injury; reduced oxidative stress; reduced Inflammation.	(118)
	H2S	Animal model: Allogeneic renal transplantation with donor rats that were flushed with cold solution or cold solution plus 150 μM NaHS (H2S group).	recovery of mithocondrial function; improved syngraft function.	(119)
	H2S	Animal model: Porcine kidneys from Donation after Circulatory Death (DCD)underwent subnormothermic machine perfusion with addition of H2S	improvement of organ function.	(120)
	H2S	Animal model: porcine DCD kidneys underwent normothermic machine perfusion with addition of H2S	controllable hypometabolic state, recovery of renal function.	(121)
	MHC siRNA	Animal model: permanent silencing of MHC antigens in transplanted rats.	reduced organ immunogenicity; reduced renal damage; reduced inflamamtion.	(122)
		**Clinical studies**		
***DGF following deceased donor kidney transplant***	P53 siRNA	Clinical studies: PhaseI-II-III (completed)	reduced DGF incidence; favorable recipient outcome.	https://clinicaltrials.gov/
***AKI and (MAKE) following cardiac surgery***	P53 siRNA	Clinical studies: PhaseI-II-III (Phase III ongoing)	reduction in the incidence of MAKE composite in the 90 days following cardiac surgery; reduction in the incidence of AKI in the 5 days following cardiac surgery; reduction in the severity and duration of AKI; overall survival.	https://clinicaltrials.gov/
***Deceased donor transplant***	NAC	Clinical study: 74 recipients were randomized to receive NAC 600mg twice a day or placebo.	higher mean eGFR throughout the first 90 days and at 1 year; lower risk of DGF.	(123)

OA, Oleanolic acid; BUN, blood urea nitrogen; sCr,serum creatinine; KIM-1, kidney injury molecule-1; LDH, lactate dehydrogenase; MDA, methane dicarboxylic aldehyde; GSH, glutathione; I/R, Ischemia/reperfusion; HDL, High Density Lipoprotein; NAC, N-Acetylcysteine; ATOR, Atorvastatin; H2S, hydrogen sulfide; MAKE, Major Adverse Kidney Events.

Similarly, another study evaluated the effects of an antioxidant compound, leutoline, a flavonoid obtained from plants, and demonstrated its renal protective effect in the same context (105).

Other interesting studies have been focused on the effects of non-polyphenolic substances, such as atorvastatin (ATOR) and N-acetylcysteine (NAC) in reducing oxidative stress and renal damage in a murine model of I/R injury (106). Cusamano G. et al. showed that the use of statin as ATOR, alone or in combination with NAC could better counteract the detrimental effects of oxidative stress damage and assure better outcome in renal recipients (106). The effects of NAC in preventing IR injury has been well documented in other experimental settings such as lung transplantation models (124, 125). Despite experience in the use of antioxidant compound in pre-clinical studies, few works demonstrated their effectiveness in clinical setting. Danilovic A. et al. investigated the effects of NAC in deceased donor transplant (123). They observed an attenuation of oxidative damage and early recovery of renal function in recipients who received NAC orally from day 0 to 7 postoperatively (123). Moreover, a metanalysis of six randomized controlled trials provided evidence of the effectiveness of NAC against contrast-related nephropathy (126) (**Table 1**).

Emerging studies evaluated the possibility to administrate antioxidant enzymes (AOEs), such as catalase and SOD and antioxidant compounds through carriers that protected both enzymes and compounds from inactivation and deterioration and improved intracellular delivery. Indeed, application of vascular immune targeting AOEs to specific endothelial epitopes has been proven to be an effective donor preconditioning method in lung transplantation model (127). Preissler G. et al. observed that the infusion of the antioxidant enzyme catalase, conjugated with a platelet/endothelial cell adhesion molecule-1 (PECAM-1) antibody to nanosized particles, before cardiac arrest, induced a decrease of endothelial dysfunction, lipid peroxidation, alveolar leakage, and edema formation in transplanted pigs (128). Therefore, the preconditioning approach with antioxidant agents might be a promising strategy to improve outcome in transplantation using organs from donors after cardiac death (127, 128). Likewise, vascular immunotargeting of catalase to lung endothelial cells *via* anti-angiotensin-converting enzyme antibodies attenuated oxidative damage in a rat model of lung I/R injury (127) (**Table 1**).

Finally, the use of liposomes as artificial vesicles for encapsulation and delivery of antioxidant compounds, such NAC and curcumin has been shown to protect respectively lung, liver and kidneys in rodent models (127).

Conclusively, the antioxidant therapies are promising approaches to protect graft before I/R injury. Given that ischemic-induced injury reaches the major detrimental effects in cold preservation period, several strategies including machine perfusion and supplemented preservation solutions with anti-oxidant compounds have been introduced to ameliorate transplant outcome.

## Strategies of Intervention

### Donor Strategies

#### Donor Pre-Treatment

Therapeutic management of donors can have a crucial impact on recipient’ outcomes reducing the effects of IRI during the early post-transplant phase (22). The main goals of donor management are the maintenance of an adequate volemia, the optimization of cardiac output and blood pressure in order to ensure adequate perfusion pressure and blood flow avoiding the use of a significant amount of vasoactive drugs (22). In a recent retrospective study, including 214 consecutive recipients from 122 brain-dead donors, meeting optimal donor management goals (DMGs) at donor neurological death is associated with a lower risk for DGF, independent of the use of machine perfusion and donor quality (129). In this scenario, donor pre-treatments have been investigated in several experimental and clinical studies with the goal of prevention and early treatment of organ injury during IRI, while only few studies analyzed their impact on graft survival (130). Donor pre-treatments are usually classified in physical (including hypothermia and remote ischemic preconditioning) or pharmacological approaches (including dopamine and steroids). Mild hypothermia has been demonstrated to significantly reduce the rate of DGF among recipients: in a randomized clinical trial including 394 donors randomized to mild hypothermia (34-35°C) or normothermia (36.5 to 37.5°C), DGF occurred in 79 recipients (28%) in the hypothermia group and in 112 recipients (39%) in the normothermia group (OR 0.62, 95%CI 0.43 to 0.92, p=0.02) (131). More recently, Schunuelle et al. have investigated the impact of spontaneous donor hypothermia (defined as a core body temperature less than 36°C 4-20 hours before organ retrieval) on DGF and 5-year graft survival: although donor hypothermia reduce di risk for DGF, a significant advantage in terms of graft survival was not described (HR 0.83, 95%CI 0.54 to 1.27, p=0.39) (132). Remote ischemic preconditioning (RIPC) has been used as a strategy to reduce acute kidney injury in the setting of cardiac surgery; while promising data suggested a potential benefit of RIC in animal kidney transplantation models, a few clinical trials have investigated the use of RIC in human kidney transplantation with controversial results. In a murine model, ischemic preconditioning (15 min of warm ischemia followed by 10 min of reperfusion) has been shown to protect from both warm and cold ischemia, increasing the local production of nitric oxide (133). In a small RCT where RIPC was performed using a pneumatic tourniquet place on both thighs for 10 minutes at the time of organ retrieval, no significant differences in eGFR or serum creatinine were described, while an increase in pro-inflammatory cytokines has been reported (134). More recently, Bang et al. investigated whether RIPC performed in living kidney donors using a blood pressure cuff placed on upper arm (3 cycles of inflation to 200mmHg for 5 minutes followed by deflation of the cuff for 5 minutes) could improve kidney function and outcomes in both donors and recipients; serum creatinine levels for donors at discharge was significantly lower in donors who received RIPC, while no significant difference in serum creatinine, eGFR, risk for DGF, acute rejection and graft failure between the recipients of the two groups (135). Several pharmacological donor pre-treatments have been recently investigated in this setting. A randomized trial that randomized 264 deceased heart-beating donors in 60 European Centers to receive pre-treatment with low-dose dopamine (4mug/kg/min) reported a lower incidence of dialysis requirement in the first week after transplantation in recipients of a dopamine-treated graft (136). However, a secondary analysis of this trial investigating the impact of dopamine infusion on long-term outcomes did not show significant advantages in 5-years graft survival, suggesting that a longer dopamine infusion time may instead improve clinical outcomes (132). In a multicenter randomized controlled trial, 306 deceased donors were randomized to receive corticosteroids (1000 mg of methylprednisolone) or placebo prior to organ procurement: the rate of biopsy-proved rejection at 3 months as well as 5-year graft survival did not significantly differ between the pre-treated and placebo groups, suggesting that this approach did not impact on long-term clinical outcomes (137).

### *Ex Vivo* Perfusion Strategies

#### Hypothermic Machine Perfusion

A great deal of research is still needed to optimize the techniques of organ transport and storage for maintaining and improving transplant outcomes. The widely used methods for hypothermic storage of deceased-donor renal grafts include static cold storage (SCS) and hypothermic machine perfusion (HMP) (138, 139).

SCS (SCS) is a process by which the preservation solution is infused into the organ and then stored statically at hypothermic temperatures; currently represents the most widely used method due to its greater availability, nevertheless HMP is to prefer for long period of kidney preservation (139, 140). The main difference between the two methods consists in the fact that HMP allows to continuously perfuse the organ with cold preservation solution probably assuring reduced I/R injury and better graft outcome (139, 140). Thus, it is relevant to determine which method is more effective in terms of post-transplant results.

First of all, it is necessary to consider the principal pathological mechanisms involved in DGF, as ischemic damage and inflammation and the benefits that static cold storage or hypothermic machine perfusion could reach to preserve transplant renal parenchyma (140). Preclinical studies showed great effects of cold pulsatile perfusion *vs* static storage due to amelioration of endothelial function and reduced pathological lesions within renal parenchyma (139).

Unfortunately, few randomized clinical trials investigated the different effects between machine perfusion and cold storage in deceased donor kidney transplantation (139). In a systematic review Bathini V. et al. analyzed nine clinical studies, comparing pulsatile perfusion and static storage, not discriminating between donation after cardiac death (DCD) and donation from a neurologically deceased donor (DBD) (141). The authors observed significant results in terms of decreased incidence of DGF in transplanted kidneys from DCD (141). They did not register positive effects in 1-year graft survival, comparing all these studies (141). Accordingly, O’Callaghan JM. et al. demonstrated that cold pulsatile perfusion decreased the occurrence of DGF, and no significant effects were observed in graft survival and primary no function (PNF) (142). Jiao B. et al. compared renal outcomes in patients receiving expanded criteria (ECD) kidneys after HMP or cold static storage in a metanalysis and they observed that HMP was associated to decreased incidence of DGF and graft survival (143). Recently, Peng P. et al. analyzed only randomized clinical trials that compared the two preservation methods, bypassing some confounding factors that characterized precedent metanalysis studies (144). They concluded that HMP significantly reduced DGF occurrence in both DCD and DBD and could increase graft survival after 3 years from transplantation (144). No significant results were obtained in terms of PNF, survival rate at 1 year, loss of graft and hospitalization (144).

Recently, several studies highlighted the role of metabolic activity for the assessment of graft quality and augmented incidence of graft survival. Interestingly, Guy AJ. et al. analyzed, through NMR spectroscopy, the metabolic profile of DGF and immediate graft function (IGF) kidneys and they observed differences in glucose concentrations after 45min and 4 hours post-perfusion (145). Interestingly, higher levels of glucose were found in IGF kidneys compared to those in DGF group, suggesting that the intake of glucose is necessary to maintain metabolic competence attenuating renal damage after I/R injury (145). Moreover, they found elevated levels of aminoacidic in perfusate of DGF organs, indicating the occurrence of tubular damage (145).

##### Hypothermic Oxygenated Machine Perfusion (HOPE)

Despite oxygen consumption is significantly decreased in hypothermic condition (at 4–10°C), there is still an important cellular metabolism. The technique of HOPE (Hypothermic Oxygenated Machine Perfusion) has been recently recognized as a powerful tool in preservation technologies for the additional value to oxygenate perfusate at a partial pressure of 60–100 kPa (146–148). In rodent and pig models, pre-treatment of ischemic grafts with HOPE appeared able to counteract endothelial dysfunction, macrophage activation and improved mitochondrial metabolic pathways (149–153). Furthermore, preliminary clinical experience as well as retrospective studies started to compare incidence in DGF and other renal function parameters in HOPE treated graft versus conventional SCS kidneys (147, 154).

Recently, Ravaioli M et al. performed the first Italian clinical trial to test the effects of HOPE in kidney and liver transplant of extended criteria donors after brain death (155). In this study the authors demonstrated the importance of mitochondrial function also in renal cells (155). Therefore, the use of oxygenated perfusion demonstrated a strong decrease of ROS production and renal impairment. Moreover, they also found that NGAL measurements in renal perfusate correlates with better transplant outcome (155). Thus, these data underline the importance to perform hypothermic oxygenated perfusion in the early phase after graft retrieval, to restore mitochondrial function, reducing waste products and ROS release and organ damage. Similarly, in a prospective case-matched pilot study, Meister FA. et al. demonstrated the safety and feasibility of HOPE in human ECD grafts, finding a decrease in renal resistance as central predictor of allograft function (147). However, no significant differences were assessed in DGF incidence.

Other research groups, in particular the team of Zurigo, demonstrated the crucial role of hypothermic oxygenated perfusion (HOPE) to improve graft quality before organ implantation (156). Initially, Dutkowski P et al. performed experimental studies in various animal models (149, 157–161); they showed less reperfusion damage in rat livers of DCD that underwent HOPE for 10 hours due to a metabolic reloading (158, 162). In large animal models, they also observed that pigs transplanted with untreated DCD grafts developed severe metabolic disorder as acidosis, systemic shock and clear histological signs of liver impairment (159). In contrast, an intervention of 1 hour HOPE successfully prevented these events and transplanted pigs showed normal levels of lactate and recovery of liver function with bile production after 2 hours from implantation (159). In addition, mitochondrial function and liver ATP content was preserved, assuring less injury during the reperfusion phase.

On basis of experimental research, they performed the first worldwide clinical application in grafts obtained from DCD donors (156). The strength of this approach is associated with the preservation of mitochondrial function indicating mitigation of I/R injury and the assessment of a new biomarker, flavin, that provides a fast prediction of liver graft function (156, 163). Flavin is a marker of mitochondrial complex I injury released from the first electron transferring mitochondrial protein during oxygenated perfusion (163). Recent studies in ischemic brain mitochondria demonstrated that the binding site of flavin in mitochondrial complex 1 determined the production of superoxide anions in normothermic conditions and the release of flavin itself (164). Dutkowski’s group showed that ischemic livers released great amount of flavin, that was easily detectable in perfusate due to its natural fluorescent properties (163). Thus, flavin is considered a higher useful biomarker of impaired metabolism and ATP-breakdown, allowing to discriminate better organs for transplantation and reduce risks for recipients (163).

Collectively, these studies have shown the potential of cold pulsatile oxygenated perfusion method to avoid the detrimental effects belonged to I/R injury reducing the incidence of DGF. Despite the decrease in the rates of DGF, the long-terms effects of HMP in recipient’s outcome are not evident and recent literature underlined significant results with the use of normothermic machine perfusion (NMP).

#### Normothermic Machine Perfusion

The preservation of renal graft at a normothermic temperature offer a great repertoire of advantages. The possibility to replicate a physiological environment at a normal (37°C) or subnormal (20-25°C) body temperature can restore cellular metabolism. This result can be reached by an inflow of human blood at 37°C, that offer a substrate for ATP production.

ATP restoration, has been considered the main strength of NMP treatment (140, 165, 166).

Cold stored kidneys are characterized by a reduction in the expression of oxidative phosphorylation and glycolysis genes indicating a reduced capacity to generate metabolic substrates in anaerobic conditions (20, 167).

On the contrary, after NMP treatment these pathway were re-activated, indicating a recovered metabolic activity (8, 11, 168).

The central potential of NMP is the opportunity to assess renal function through the measurement of multiple perfusion and biochemical parameters during preservation (i.e renal resistance, urine production), to evaluate cell viability of the graft (by rising levels of lactate, aspartate aminotransferase and ATP) before than transplantation. Secondly, by keeping physiological function, these strategies may prevent deterioration of the organ (169). In addition, compared to HMP approach, NMP platform is optimal for the direct administration of therapies to target IRI or acute rejection by allowing constitutive cellular interactions, binding active compounds to target sites and keeping drugs pharmacokinetic at body temperature. The possibility to create a window for testing and functionally improve the graft can be summarized in the 4Rs of machine perfusion: Resuscitation, Repair, Rejuvenation, Regeneration (170).

##### Perfusate Composition

The NMP treatment required a well-studied combination of perfusate components that are crucial to ensure the adequate transport of oxygen and nutrients to keep cellular integrity and vascular processes during perfusion. Regarding the oxygen carrier, it that can be natural such as blood or an artificial-based medium.

In order to mimic the physiologic renal IRI, pre-clinical *ex vivo* perfusion experiments have been carried out with whole blood. However, in the human clinical setting an NMP perfusate could never be composed of whole blood (171). Firstly, human whole blood is not easily available. Secondly, the plasma and buffy coat components are rich of antibodies, coagulation mediators, leukocytes and thrombocytes. On other words, blood-derived solutions are lesser used for their tendency to haemolysis, platelet activation and long-term decreased efficiency of oxygen-carrying capacity of erythrocytes. Furthermore, after warm ischemia time, the reperfusion period is profoundly characterized by cytokine storm, complement and coagulation activation (**Figure 2**) in which leucocytes, endothelial cells and platelets play a detrimental role. Leucocyte infiltrate into the renal parenchyma leading to microvascular and glomerular congestion. The ROS released by activated macrophages further amplify renal damage, activate complement system that sustained injury response. During reperfusion, platelets led to thrombus formation, reducing flow rate and mediating also vasoconstriction and inflammatory processes (4, 14, 168, 172).

Based on these observations, solutions with packed red blood cell in the absence of leucocytes and platelets have been preferentially developed and were found to moderate infiltration, inflammatory response by improving circulation and renal function (140).

In completely artificial solutions, Brasile et al. set an acellular normothermic solution based on a perfluorocarbon (PFC), an inert compound that have the high capacity to dissolve oxygen enriched with energic substrates such as culture-like medium containing amino acids, lipids and sugars (166, 173).

From the initial generation of PFC solutions, other compositions have been developed with improved stability, even if still correlated with substantial manufacturing and costs (174).

Other artificial haemoglobin-based oxygen carriers have also been developed as Lifor, an artificial preservation medium containing a nonprotein oxygen carrier and AQIX-RS-I that resembles physiological ionic concentrations, osmolarity and ion conductivity, to maintain the cell membrane and enzymatic processes.

Finally, Hemarina- M101 is an extracellular haemoglobin derived from a marine invertebrate formulated into an oxygen carrier called Hemoxycarrier used in cold storage solutions to deliver oxygen (174).

Beside oxygen carrier, a NMP solution should also contain a colloid and a balanced electrolyte composition (175).

In the blood vessels, albumin represents the central component that keep a normal colloid osmotic pressure (176) and at glomerular level is essential to maintain a physiological ultrafiltration rate.

Several NMP solutions for kidney preservation are enriched with albumin. Other additives include glucose, insulin, aminoacids, bicarbonate for the pH, antibiotics to prevent bacterial infection and mannitol to maintain eritrocytes viability.

However, despite encouraging results, today we have a limited understanding of the precise formulation of an NMP solution. There is no an ideal perfusate and the composition appear to be strictly dependent from duration of NMP, type of graft damage (DCD, ECD) and need for regeneration and repair as well as recipient characteristics.

##### NMP Treatments

An increasing number of clinical and experimental studies provided evidence on the superiority of kidney preservation of a short treatment of NMP compared to a longer SCS (177–179). A series of seminal studies showed that a 60‐minute period of NMP after SCS made it possible to transplant a DCD kidney with greatly improved immediate graft function compared with SCS alone (180, 181).

The molecular mechanisms of NMP induced protection are still poorly investigated. In a pig model, Stone et al, demonstrated that 6h of NMP induced a pro-inflammatory cytokines storm as assessed by increased IL-6, IFN-γ, and CXCL-8 that was able to induce the mobilization of donor-derived leukocyte, then their removal prior to kidney transplantation (150). Similar results were also found in other porcine studies from Hosgood SA et al. who demonstrated that kidneys undergoing a short period of NMP had increased expression of heat shock protein 70 and IL-6 together with improved metabolic function and less tubular injury compared with static cold-stored kidneys (182). From the other side, these pro-inflammatory pathways were also downregulated during cold storage, including TNFα activation *via* NF-kB and reactive oxygen species pathways. These results could appear confounding in the perspective of a protective and beneficial effect of NMP compared to SCS. However, it’s well accepted that these early, pro-inflammatory processes help to condition the kidney in preparation for reperfusion and that cold storage merely represents a transitory hold on these pathways. Therefore, following reperfusion in the recipient, similar changes in inflammatory gene expression as observed in NMP are expected. As demonstrated by Hameed AM et al, by performing transcriptomic analysis of NMP in three kidneys undergoing NMP, authors found the induction of immune response-related genes during NMP, including IL1B, CXCL2, and TNF. However, a short NMP treatment of 1h after cold ischemia was able to activate protective stress responses, promoted cell survival and graft recovery (169). Similar results were provided by other groups that evaluated the release of cytokines, chemokines and donor leukocytes from the interstitial compartment of the kidney into the perfusate during NMP as a source of inflammation. The combination of filters into the circuit could reduce cytokines to shut down IRI associated inflammatory state.

Ferdinand JR et al. (183) by whole kidney transcriptome analysis compared the effect of NMP with that of cold storage by using pairs of human kidneys obtained from the same donor. Firstly, authors confirmed that cold storage led to a comprehensive reduction in gene expression of inflammatory and metabolic signalling such as oxidative phosphorylation. By contrary, during NMP, there was marked increase of oxidative phosphorylation genes, and not surprisingly, also of immune and inflammatory pathway genes. Retrospectively, considering that NMP-treated grafts were transplanted, authors found that higher inflammatory gene expression occurred in organs with extended DGF and that the use of a hemoadsorber significantly modulated the expression of a DGF-associated gene signature.

Despite the development of different devices, the basic aspects of NMP procedure is the same with four common components: an oxygenator, a blood reservoir, a pump for renal artery inflow and a heat exchanger.

However, at present there is no consensus of an ideal protocol of reperfusion. NMP can be realized by a hugeness of approaches ranging from duration of reperfusion, pressure, continuous or pulsatile flow, temperature, partial pressure of oxygen, sequence with cold static storage and type of perfusion solutions (184). As a consequence, the clinical applications of NMP are strongly influenced by the parameters applied in the centre. Furthermore, the NMP experience in clinical setting is sparse, with only several case reports (185).

The first group to translate the preclinical experience into clinical reality was Hosgood et al. by transplanting a 62-year-old ECD kidney into a 55-years old recipient. Kidney was subjected to 11 h of SCS, then perfused with a red cell-based solution at 33.9°C for 35min, then transplanted. Despite a short delay in recovering the graft function, patient remained dialysis free with a serum creatinine level of 132 μmol/L at 3 months post-transplantation. In contrast, in the recipient of the contralateral kidney the DGF occurrence was observed (186). Moreover, in another study, eighteen kidneys from ECD were subjected to a period of NMP by plasma free red-cell based solution at a mean temperature of 34.6°C. Authors compared outcome of these kidneys to a control group of ECD kidneys subjected to conventional SCS. Strikingly, also in this cohort, incidence of DGF was substantially decreased in the NMP group (5.6 *vs.* 36.2%), even if no differences were observed in patient survival at 12 months or acute rejection incidence (177).

To date, performed clinical studies have assessed short‐term durations of perfusion (1‐2 hours), with the aim to restore cellular metabolism and counteract immune inflammation before reperfusion in the recipient. Brasile et al. described successful 48h perfusion of isolated canine and human kidneys. More recently, Weissenbacher et al. was able to maintain the quality of discarded kidneys from DBD and DCD donors for up to 24 h. These prolonged strategies allow clinicians to retrieve time for viability and functional assessment, as well as for implementation of perfusate with new drugs (176, 187, 188). Although the primary results are encouraging, more research focusing on the reduction of immunogenicity of ECD organs is needed. A randomized, controlled multicentre UK trial of DCD kidneys is underway to assess the effects 1h period of NMP compared to static cold storage with the result expected in 2021 (ISRCTN15821205) (189).

All these studies compared NMP to SCS, a limited number of research article are available on the comparison between NMP and HMP (190–193). In these porcine models, an overall improvement in renal function was demonstrated in NMP compared to HMP, even if Darius et al. found that a continuous oxygenated period of HMP from time of retrieval to be better that NMP and Blum et al. described similar renal functionality after an 8 hour period of HMP or NMP (192, 194).

Recently, in a swine model of renal transplantation, Vallant et al. directly compared kidneys pairs from the same donor and after 4h of HMP followed by 2h of simulated reperfusion by NMP with whole blood. They demonstrated enhanced renal resistance, better renal histology in HMP group compared to NMP. Intriguingly, authors used healthy, young, slim donor pigs, reducing potential external influencing factors to a minimum, however they performed only two hours of simulated reperfusion, thus a relatively short time to investigate long-term effects of renal grafts.

### *Ex Vivo* Therapeutic Strategies

The use of drugs in preservation solution represents the “new era” of therapeutic strategies designed to reduce I/R injury, exerting antioxidative, anti-inflammatory, and anti-apoptotic activities.

From the flavonoid group of polyphenols, Quercitin (Que) has been reported to possess strong anti-inflammatory and antioxidant properties, attenuating inflammation and apoptosis (195, 196). Several studies analyzed the effects of Que and sucrose (Scr) in CS solution, underling their effects in reducing tubular damage, inflammatory infiltrate, limiting the I/R injury (111, 115) (**Table 1**).

Recently, Gochi M et al. showed the effects of Que and Scr in CS solution and hypothermic oxygenated perfusion (HOPE) in autologous transplantation models (116). They found that the addition of Que and Scr in preservation solution ameliorated renal function and reduced oxidative stress, preserving renal parenchyma (116). Despite these strong results, this study did not demonstrate that Que could exert beneficial effects in long term-outcome (116). Therefore, clinical studies should be performed to investigate the effects of Que in terms of graft survival and recipient outcome.

Another renoprotective drug that has attracted the attention of researchers is the Metformin (197). It is widely used as antihyperglycemic drug to treat patients with type 2 diabetes (198). The mechanism of action is still not completely clarified, but several studies reported pleiotropic effects in inhibition of the complex 1 of the mitochondrial respiratory chain (198, 199). Beyond its principal effects in glucose-decrease, this drug could reduce endothelial dysfunction, inducing nitric oxide production, activating cellular energy pathways, and reducing the expression of some inflammatory markers as Endothelin 1 (ET-1) (200, 201). During renal I/R injury, ET-1 has widespread effects on renal parenchyma and increased levels are associated with damage, endothelial dysfunction and fibrosis (202) (**Table 1**).

Moreover, animal studies have reported that pre-treatment with metformin could reduce both myocardial and cerebral I/R injury (203, 204). However, no significant results were obtained in randomized clinical studies in myocardial setting (205, 206). Recently, Huijink T M et al. tested the effects of metformin in a rat model of renal I/R injury and in NMP with ischemic porcine kidneys (112). In rat experimental model, pre- and post-conditioning with metformin significantly reduced tubular cell necrosis and endothelial dysfunction and improved organ quality (112). However, no significant differences were observed comparing pre and post conditioning effects. In porcine study, creatinine clearance did not differ between kidneys perfused in NMP with metformin and without drug, even if there was a tendency toward lower creatinine levels in metformin-treated group (112). Histological analysis of porcine renal biopsies reported no differences in morphological lesions of renal parenchyma between two NMP groups (112). Therefore, metformin can be used in renal transplantation setting but it remains unclear whether addition of this drug during machine perfusion results in improved organ quality after transplantation (112).

Recently, Sedigh A et al. evaluated the efficacy of heparin when added in HMP modality to reduce renal damage in a swine model of I/R injury (114). They observed that heparin bound endothelial cells of glomeruli capillaries, probably promoting a first line of protection to preserve filtration function (114). Moreover, there was a great decrease of renal parenchymal damage and a lower intrarenal resistance that could be associated with a better outcome of the graft. Beyond anticoagulant properties, heparin has anti-inflammatory functions and could attenuate renal I/R injury (113). Therefore, the addition of heparin in machine perfusion approach could be useful in implementing cadaveric organ quality (113, 114) (**Table 1**).

Some evidences suggested that the protective role of HDL against I/R injury is not limited to the cardiac muscle but could be extended to other organs and tissues (207). In particular, Thiemermann C et al. evaluated the effects of HDL in a rat model of renal I/R injury (110). Since inflammatory process represents a key mechanism in renal I/R injury, the use of HDL confirmed less renal dysfunction with reduced serum creatinine levels and recovery of glomerular, endothelial and tubular function (110) (**Table 1**). Recent studies demonstrated that HDL could prevent I/R injury also in brain, intestine, liver, and lung (207). Therefore, it is clear that if administered promptly in donors or in machine perfusion system could prevent organ damage and probably assure a better recipient outcome.

#### Delivery of siRNA

The kidney may represent a specific target for RNA interference therapy, considering its structure and higher vascularized parenchyma (208).

Several clinical studies have been performed to evaluate the effects of p53 siRNA in reducing I/R, preventing AKI and DGF (209–211). The first trial was a Phase I, randomized, double-blind study that was designed to reduce p53 levels decreasing the incidence of AKI during cardiovascular surgery (208) (**Table 1**). In the last years, Quark Pharmaceuticals pushed the use of this compound in deceased donor kidney transplant to avoid the occurrence of DGF (208). From the Phase I to Phase III, overall results demonstrated reduction in DGF incidence and severity and favorable recipient outcome. In May 2018, a phase III multi-center trial is designed to evaluate p53 siRNA versus placebo for the prevention of Major Adverse Kidney Events (MAKE) in subjects at high risk for acute kidney injury following cardiac surgery (208). To date, this study is ongoing but the Food and Drug Administration (FDA) could expand its use in transplant field.

Yang C. et al. performed several experiments to test the effect of caspase-3 siRNA in cold preservation solution and in swine auto transplantation model (117). In their first experiments, they observed significant protective effect only in *ex vivo* perfusion model (212). Optimizing naked caspase-3 siRNA stability by chemical modification, they demonstrated a significant improvement of renal function also in auto transplantation model (117) (**Table 1**).

Another important key mechanism in renal I/R injury is represented by complement system activation. Treatment with C5a siRNA preserved renal parenchyma in a mouse model of I/R injury (107). In addition, Zheng X et al. demonstrated that the use of both C3 and Caspase-3 siRNAs decreased complement activation (108). Local inflammatory response, infiltration of inflammatory cells, parenchymal damage with recovery of renal functions. Recently, de Ramon L et al. demonstrated that siRNA inhibition of CD40, a costimulatory mediators of T cells response, strongly improved renal inflammatory status in a rodent transplantation model (109) (**Table 1**).

Finally, Yuzefovych Y et al. highlighted the potential of siRNA approach in *ex vivo* perfusion system of donor organs by downregulating major histocompatibility complex (MHC) class I and II transcripts in transplanted graft (122). Despite the advances for crossing the HLA barrier in transplantation, the use of immunosuppressive drugs is necessary to achieve graft long-term outcomes, but it is accompanied by reduced immunity to infection and malignant diseases (122) (**Table 1**). Therefore, targeted MHC siRNA could aid to reduce organ immunogenicity and offers the possibility to overcome the problems related to immunosuppression therapy and obtain a better recovery of the graft (122).

#### H2S Supplementation

An emerging candidate with potential effects in I/R field is the biological agent named hydrogen sulfide (H2S). It is an endogenous molecule released by gas transmitter that exerts cytoprotective effects ranging from anti-oxidant to anti-inflammatory and anti-apoptotic properties (118) (**Table 1**).

In the context of allogeneic renal transplantation, Lobb I et al. demonstrated that the use of University of Wisconsin preservation solution plus NaHS modulated the allograft transcriptome, reducing the expression of genes involved in inflammation, apoptosis, oxidative stress and coagulation (118). In accordance, the same authors observed that H2S treatment prevent mitochondrial dysfunction and could represent a therapeutic strategy to reduce graft injury associated with prolonged cold I/R injury (119).

Recently, Juriasingani S et al. evaluated the effects of H2S in sub normothermic static solution, using *ex-vivo* DCD porcine kidneys (120). After 24h from H2S supplementation, kidneys showed lower tubular necrosis and overall damage. Another study employed H2S to supplement preservation solution in NMP (121). The addition of H2S induced a reversible hypometabolic state during NMP reducing ROS levels and renal lesions, potentially avoiding the deleterious effects of I/R injury (121).

All these studies support the use of H2S for clinical purposes considering its effects in inducing reversible state of hypometabolism without functional or structural deterioration. More research is needed to determine long term effects of H2S and its use in the transplantation setting.

## Recipient Strategies: Treatment to Counteract IRI

Although donor pre-treatments have been investigated with controversial results, an optimal management of the recipients may minimize the impact of IRI in kidney transplantation (22). It is strongly recommended to avoid dehydration before transplantation in order to reduce the risk of hypovolemia: the central vein pressure should be maintained higher than the usual normal range for an adequate kidney perfusion (22). As recommended by European Renal Best Practice guidelines, the maintenance of an optimal intravascular volume using isotonic crystalloids rather than colloids and, when possible, the exclusion of any potential nephrotoxic drugs may reduce the risk of worsening renal function in the early period after kidney transplantation; in this scenario, treatment with calcineurin inhibitors may be postponed after kidney function improvement (213). In addition, antioxidant treatment may be considered in reducing the risk of IRI: in a small clinical study where 74 recipients were randomized to receive N-acetylcysteine, 600mg twice a day, or placebo, the treated group showed a higher mean eGFR throughout the first 90 days and at 1 year, and the risk of DGF was significantly lower (123).

## Conclusion and Future Perspectives

Regardless donor type, IRI is an unavoidable consequence after kidney transplantation that could lead to graft failure.

The last decade has been characterized by huge efforts in renal hypothermic and normothermic *ex-vivo* machine perfusion techniques, mainly to face the severe shortage of organs available for transplantation. *Ex vivo* HMP and NMP treatment offer a window of opportunity for testing and improve suboptimal graft, summarized in the 4Rs: Resuscitation, Repair, Rejuvenation, Regeneration. Organs with greater ischaemic insults could be successfully utilized leading to the approval of more uncontrolled DCD organs. ECD kidneys could be treated with protective agents during perfusion to reduce the detrimental impact of IRI. The comprehension of molecular mechanisms of renal IRI and DCD donation together with the application of new preservation technologies has led to experimental setting of new drugs to be delivered in the perfusion phase. This area of research has the enormous potential to develop a list of perfusion solutions enriched with different compounds able to block precisely oxidative stress, inflammaging, complement activation or immune response based on clinical observations.

Ideally, a personalized, organ-tailored formulations could be chosen based on degree of ischemic damage, the condition of the donor or the recipient, the expected duration of the treatment or the donor age.

However, to date, the exact formulation of a perfusion solution is still unknown, there is no an ideal composition and we are still far away from predicting long-term renal post-transplantation outcome based on resistance or other parameters collected from perfusion devices.

The diffusion of new perfusion technologies, the publication of international registry to standardize perfusion protocols and retrieve data will permit to anticipate renal outcome. Finally, the application of omics studies (from transcriptomic to proteomics) would lead to identification of new biomarkers to be monitored during perfusion and in the recipient.

## Author Contributions

Conceptualization, writing, and editing of the manuscript by RF. AS and MF contributed to the writing and drafting of the work. LG, GS, RO and GC contributed substantially to the work by critical revisions and draft editing. LG and SS revised the work critically for important intellectual content. All RF conceived figures. All authors have read and agreed to the published version of the manuscript. All authors contributed to the article and approved the submitted version

## Funding

This work was supported by the University of Bari ‘Aldo Moro’, Ministry of Education, University and Research (European Union– European Social Fund, PON R&I 2014–2020, Azione I.2 “Attrazione e Mobilità Internazionale dei Ricercatori”-AIM 1810057-activity 2 granted to AS and to SI-Ca.Re. (Integrated system for monitoring and care of patients with Cardio-Renal syndrome) - New model of hospital-territory integration for the home care of patients suffering from heart failure and chronic renal failure. (INNONETWORK Call - Puglia Region).

## Conflict of Interest

The authors declare that the research was conducted in the absence of any commercial or financial relationships that could be construed as a potential conflict of interest.
